# Prevalence and genetic diversity of *Anaplasma* and *Ehrlichia* in ticks and domesticated animals in Suizhou County, Hubei Province, China

**DOI:** 10.1038/s41598-024-63267-6

**Published:** 2024-06-01

**Authors:** Ju Tang, Jiao Xu, Xiao-hui Liu, Fang-zhi Lv, Qiu-ju Yao, Xiao-Fan Zhou, Hui-ya Lu, Tian-mei Yu, Ze-zheng Jiang, Xiao-zhou Jin, Fang Guo, Xue-jie Yu

**Affiliations:** 1 State Key Laboratory of Virology, School of Public Health, Wuhan University, Wuhan, Hubei China; 2 Suizhou Center for Disease Control and Prevention, Suizhou, Hubei Province China

**Keywords:** *Anaplasma*, *Ehrlichia*, Ticks, Goats, China, Epidemiology, Infectious diseases

## Abstract

*Anaplasma* and *Ehrlichia* are tick-borne bacterial pathogens that cause anaplasmoses and ehrlichioses in humans and animals. In this study, we examined the prevalence of *Anaplasma* and *Ehrlichia* species in ticks and domesticated animals in Suizhou County, Hubei Province in the central China. We used PCR amplification and DNA sequencing of the 16S rRNA, *gro*EL, and *glt*A genes to analyze. We collected 1900 ticks, including 1981 *Haemaphysalis longicornis* and 9 *Rhipicephalus microplus*, 159 blood samples of goats (n = 152), cattle (n = 4), and dogs (n = 3) from May to August of 2023. PCR products demonstrated that *Anaplasma bovis*, *Anaplasma capra*, and an *Ehrlichia* species were detected in the *H. longicornis* with the minimum infection rates (MIR) of 1.11%, 1.32%, and 0.05%, respectively; *A. bovis*, *A. capra*, and unnamed *Anaplasma* sp. were detected in goats with an infection rate of 26.31%, 1.31% and 1.97%, respectively. *Anaplasma* and *Ehrlichia* species were not detected from cattle, dogs and *R. microplus* ticks. The genetic differences in the *gro*EL gene sequences of the *Anaplasma* in the current study were large, whereas the 16S rRNA and *glt*A gene sequences were less disparate. This study shows that ticks and goats in Suizhou County, Hubei Province carry multiple *Anaplasma* species and an *Ehrlichia* species, with relatively higher infection rate of *A. bovis* in goats. Our study indicates that multiple *Anaplasma* and *Ehrlichia* species exist in ticks and goats in the central China with potential to cause human infection.

## Introduction

*Anaplasma* and *Ehrlichia* are tick-borne bacterial pathogens causing anaplasmoses and ehrlichioses respectively in humans and animals^1–3^. The pathogens have a high degree of biodiversity and a wide geographical distribution, posing a serious threat to humans and domesticated animals worldwide. At present, the genus *Anaplasma* comprises *A. phagocytophilum*, *A. bovis*, *A. capra*, *A. centrale*, *A. marginale*, *A. ovis*, and *A. platys*^4^.

*Anaplasma phagocytophilum* was first identified in 1932 as a tick-borne pathogen, which can infect the neutrophils of rodents and ruminants, such as cattle and sheep, with fever, malaise, headache, myalgia, leukopenia and thrombocytopenia^5^. Among healthy populations, Africa has the highest seropositivity rate for *A. phagocytophilum* (21.7%), followed by Asia (3.30%–27.08%), Europe (0%–16.28%), and the Americas (0%–7.75%)^6^. *Anaplasma bovis*, which normally parasitizes mononuclear cells, was first detected in cattle in Brazil in 1936. Its primary host is cows, buffaloes, goats, and sheep. Recent studies have shown the presence of *A. bovis* in the blood and lung tissue of horses^7,8^. *Anaplasma capra*, recently discovered in China, can infect a wide range of ruminants, including goats, sheep, and deer, as well as humans^9^.

*Ehrlichia* spp. are intracellular bacteria found in the cytoplasmic vesicles of monocytes, granulocytes, or platelets in humans and animals, causing fever, leukopenia, and thrombocytopenia^10^. In the genus *Ehrlichia*, *E. ruminantium* and *E. minasensis* can infect cattle. *Ehrlichia ruminantium* can also infect other ruminants like sheep, goats, and buffalo; *E. chaffeensis*, *E. ewingii*, *E. muris*, and *E. canis* have been reported to infect humans^11^.

*Anaplasma* and *Ehrlichia* are a group of zoonotic pathogens, and ticks play a very important role in transmitting the pathogens. Ticks can transmit the pathogens to a wide range of domesticated animals such as dogs, goats, sheep and bovines and also to humans, resulting in anaplasmoses and ehrlichioses respectively. *Haemaphysalis longicornis*, a hard body tick, is distributed throughout China and can infest on a wide range of host animals such as dogs, goats, sheep, and cattle^1,12–14^. *Anaplasma capra*, *A. bovis*, *A. ovis*, *A. phagocytophilum*, and *A. marginale* have all been reported in *H. longicornis* in China^4,15,16^. In Hubei Province, China, *H. longicornis* have been found to transmit *Ehrlichia* sp., *A. marginale* and *A. bovis*. *Rhipicephalus microplus* have been found to transmit *E. canis*, *A. marginale*, *A. capra* and *A. platys* from Wuhan and Huangshi cities of Hubei Province^17^.

The aim of this study was to investigate the prevalence and genetic diversity of *Anaplasma* and *Ehrlichia* in ticks and domesticated animals collected from Suizhou County, Hubei Province.

## Materials and methods

### Ethical statement

The collection of ticks and animals’ blood for microbiological studies was approved by the Ethics Committee of the Medical School, Wuhan University (2020YF0051), and all efforts were made to minimize discomfort to the animals. We confirm that all methods were carried out in accordance with Laboratory animal—Guideline for ethical review of animal welfare.

### Sample collection and processing

**Figure 1:**
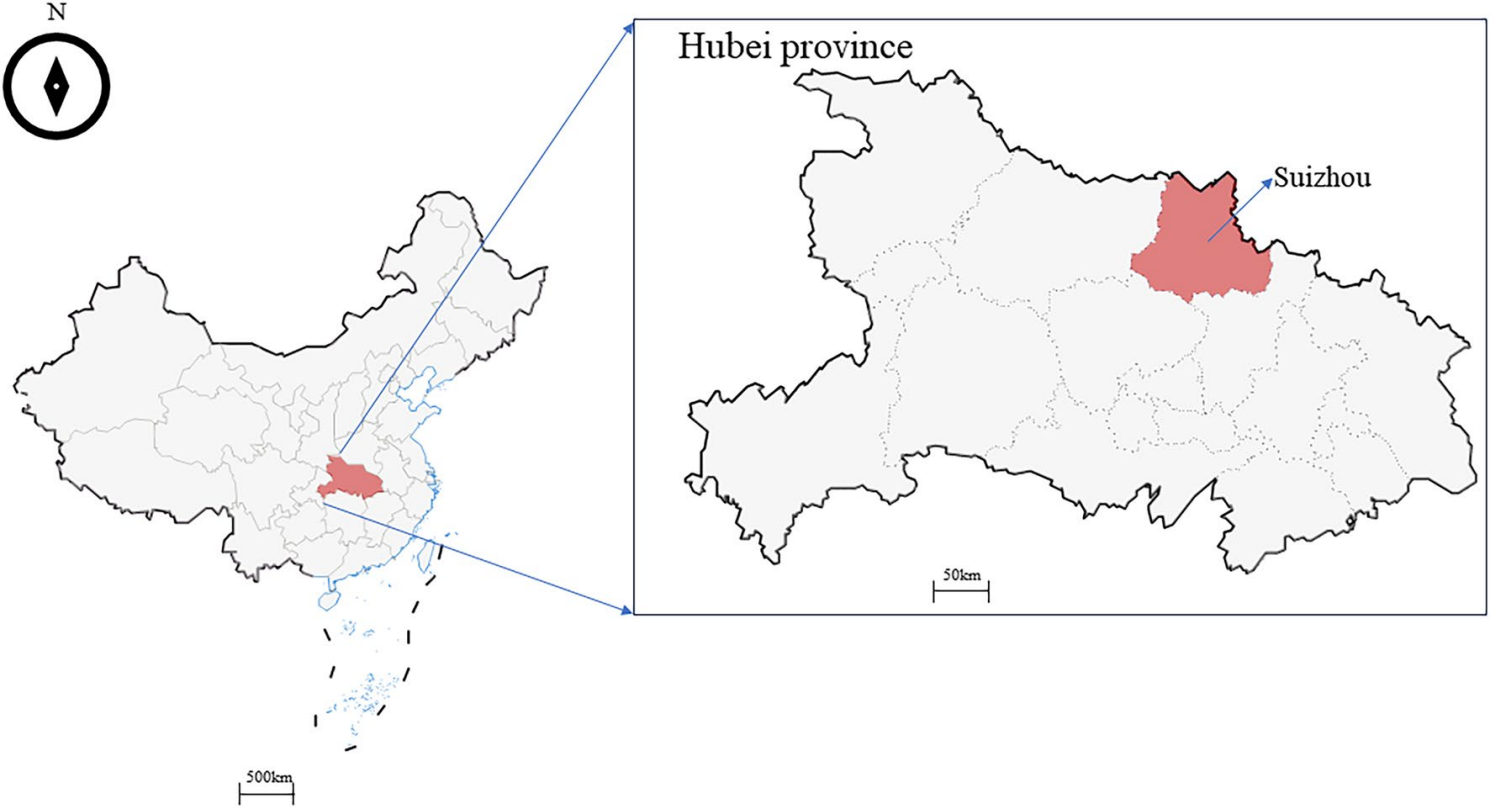
Maps showing the location of Suizhou County, Hubei Province in China, where ticks and domesticated animals’ blood were collected.

From May to August, 2023, ticks and blood samples of domesticated animals were collected from Suizhou County (113.82°E, 31.61°N), Hubei Province in central China (Fig. 1). Suizhou County is located in the northern part of Hubei Province, which is a hilly area with low mountains. It belongs to subtropical monsoon climate, with warm and humid climate, average annual rainfall of 865–1040 millimeters, and average annual temperature of 15.5 °C. There are 37 towns in Suizhou County, eight towns were randomly selected as sampling sites through simple random sampling method, and three grazing areas were randomly selected as sampling areas in each selected town. Blood (1–2 mL) was collected from the jugular vein of goats and cattle and from the cephalic vein of dogs with a 5 mL syringe and stored in EDTA anticoagulant tube. The blood samples were placed on ice and transported to the laboratory. Tick species were identified morphologically under a stereo microscope (Phenix, Shangrao, China) with a taxonomic key^18^, and representative tick species were confirmed with PCR amplification and sequencing the 16S rRNA gene (*rrs*) with primers listed in Table 1. The amplified sequences were compared with tick sequences in the GenBank with BLAST (https://blast.ncbi.nlm.nih.gov/Blast.cgi) to obtain tick species.

**Table 1:** Primers used for tick species identification and screening of tick-borne pathogens.

Species	Gene name	PCR method	Primer name	Primers sequence	PCR product length (bp)	Annealing temperature (°C)	Reference
Tick	Mitochondrion *rrs*	PCR	mt-rrs-F	CTGCTCAATGATTTTTTAAATTGCTGTGG	460	55	^19^
			mt-rrs-R	CCGGTCTGAACTCAGATCAAGTA			
*Anaplasma*	*rrs*	Nested PCR	EC9	TACCTTGTTACGACTT			^20^
			EC12A	TGATCCTGGCTCAGAACGAACG			
			EM87F	GGTTCGCTATTAGTGGCAGA	477	51	
			EM584R	CAGTATTAAAAGCCGCTCCA			
*Anaplasma/Ehrlichia*	*groEL*	Nested PCR	gro607F	GAAGATGCWGTWGGWTGTACKGC	730	57	^21^
			gro1294R	AGMGCTTCWCCTTCWACRTCYTC			
			gro677F	ATTACTCAGAGTGCTTCTCARTG	364	53	
			gro1121R	TGCATACCRTCAGTYTTTTCAAC			
*A. capra*	*glt*A	Nested PCR	Outer-f	GCGATTTTAGAGTGYGGAGATTG	793	53	^9^
			Outer-r	TACAATACCGGAGTAAAAGTCAA			
			Inner-f	GGGTTCMTGTCYACTGCTGCGTG			
			Inner-r	TTGGATCGTARTTCTTGTAGACC			
*A. bovis*	*glt*A	PCR	Abov_gltA2F	CGGAAATTACTT TTATAGATG G	826	49	^22^
			Abov_gltA2R	CATACCAYTGAG AAACCCAAC			
*Ehrlichia*	*rrs*	Nested PCR	Eh-out1	TTGAGAGTTTGATCCTGGCTCAGAACG	650	55	^23^
			Eh-out2	CACCTCTACACTAGGAATTCCGCTATC			
			Eh-gs1	GTAATAACTGTATAATCCCTG	280	55	
			Eh-gs2	GTACCGTCATTATCTTCCCTA			

**Figure 2:**
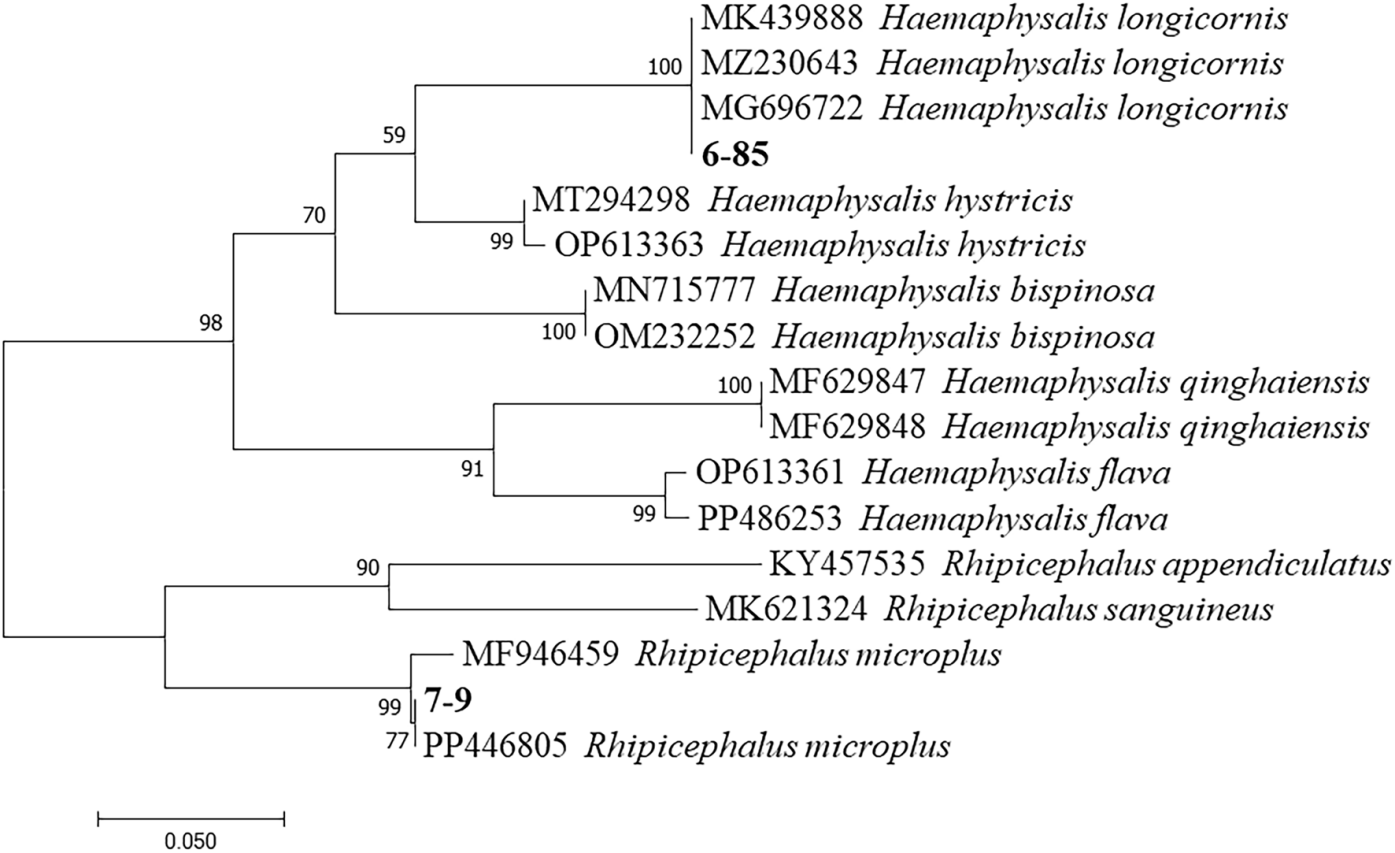
Phylogenetic tree of the tick species based on tick mitochondrion *rrs* sequences (380 bp). Sequences from this study are bolded.

A total of 1900 ticks were collected in this study. *Haemaphysalis longicornis* (n = 1891, 99.53%) and *R. microplus* (n = 9, 0.47%) were identified by morphology and molecular biology (Fig. 2). Ticks were collected from grassland (n = 932, 49.05%) and from animals’ bodies (n = 968, 50.95%). The ticks from animals were further categorized according to the degree of blood sucking as fully engorged ticks (n = 57, 3.00%), partially engorged ticks (n = 298, 15.68%), and unfed ticks (n = 1,545, 81.32%). A total of 152 goats’ blood samples, 4 cattle blood samples, and 3 dogs’ blood samples were collected.

### DNA extraction and PCR amplification of tick-borne bacteria

In order to facilitate sample processing and reduce the number of samples for DNA extraction, ticks were pooled together according to their life stage and feeding status. Each group consisted of 20 larvae, 20 nymph ticks, 10 non-engorged adult ticks, or 1 engorged tick. Ticks were rinsed three times with 70% ethanol and distilled water for 5 min each time, and air-dried. After freezing with liquid nitrogen, ticks were homogenized with metal beads using Restsch MM400 mixer mill (Retsch, Haan, Germany). Tick DNA and animal whole blood genomic DNA (gDNA) were extracted using the Trelief™ Animal Genomic DNA kit (Tsingke, Beijing, China) following the manufacturer’s instructions. The DNA concentration and quality were assessed using QIAxpert (Qiagen, Hilden, Germany). DNA samples were stored at –40 °C.

16S rRNA, *gro*EL and *glt*A gene fragments of *Anaplasma* and *Ehrlichia* were amplified with primers described in Table 1. Negative controls were set in each PCR assay with nuclease-free water as template. The amplification was performed as follows: 95 °C for 3m, 35 cycles of 95 °C for 30 s, annealing for 30 s, and 72 °C for 30–60 s, and a final extension at 72 °C for 10m. Annealing temperatures are presented in Table 1. The PCR condition was applied to all genes’ amplification in this study. PCR products were analyzed with a 1–1.5% agarose gel, stained with GoldviewTM nuclear staining dyes (GL biotech, Shanghai, China), and visualized under UV light. DNA extraction, fragment amplification, and agarose gel electrophoresis were performed in separate rooms to prevent PCR contamination. The expected PCR bands were excised from gel and purified using DNA Gel Extraction Kit (Tsingke, Beijing, China). The gel-purified DNA was ligated into the pMD 19-T vector (Takara Bio, Dalian, China) and transformed into DH5α competent cells of *Escherichia coli* for cloning. At least three positive clones were sequenced with universal primers M13–47/M13–48 for each sample. DNA sequencing was performed by Sangon Biotech Company (Shanghai, China). The company used an Applied Biosystems™ 3730XL sequencer from Thermo Fisher.

### Phylogenetic analyses

DNA sequences from this study were first analyzed using Seqman Pro (www.dnastar.com) (DNASTAR, Madison, WI)^24^ and then were compared to the sequences in the GenBank with the BLAST program. After removing the primers regions, sequences from this study, together with the reference sequences from the GenBank were imported into MEGA 11.0 software (https://www.megasoftware.net), and aligned with ClustalW (Selecting the default parameters). Only high-quality sequences with a single peak distribution of all bases and with overlapping forward and reverse sequences were used for phylogenetic analysis. Phylogenetic analysis was performed using the Maximum Likelihood method, Kimura 2-parameter model^25^ with 1000 bootstraps in MEGA 11.0 (other parameters are default). The phylogenetic results were exported to Microsoft Office for phylogenetic tree landscaping and editing.

## Results

### Prevalence of *Anaplasma* and *Ehrlichia* in ticks and animals

The DNA samples were first amplified with *Anaplasma* and *Ehrlichia gro*EL universal primers. The DNA sequences of the PCR products were analyzed with BLAST to determine which species the DNA sequences belonged to. The infection rate of *Anaplasma* in *H. longicornis* was 2.43% (46/1891) including 1.11% *A. bovis* (21/1891), 1.32% *A. capra* (25/1891), and the infection rate of *Ehrlichia* in *H. longicornis* was 0.05% (1/1891). Neither *Anaplasma* nor *Ehrlichia* was detected in *R. microplus* (0%, 0/9). The infection rate of *Anaplasma* in goats was 29.60% (45/152), including 26.31% *A. bovis* (40/152), 1.31% *A. capra* (2/152), and 1.97% an unnamed *Anaplasma* sp. (3/152) (Table 2). No goat was positive to *Ehrlichia*; neither cattle nor dogs were positive to *Anaplasma* or *Ehrlichia*.

**Table 2:** Prevalence of *Anaplasma* and *Ehrlichia* species in ticks and domesticated animal blood samples collected in Hubei, China.

Host	Pathogen species	Infection rate (%)
*Haemaphysalis longicornis*	*Anaplasma bovis*	1.11 (21/1891)
	*Anaplasma capra*	1.32 (25/1891)
	*Ehrlichia* sp.	0.05 (1/1891)
Goats’ blood samples	*Anaplasma bovis*	26.31 (40/152)
	*Anaplasma capra*	1.31 (2/152)
	*Anaplasma* sp.	1.97 (3/152)

The *gro*EL PCR-positive tick DNA was further amplified with *Anaplasma* and *Ehrlichia rrs* primers and *A. bovis* and *A. capra glt*A species-specific primers. Of the 47 *gro*EL positive *H. longicornis* samples, 33 samples were positive with *rrs*, including *A. bovis* (10), *A. capra* (22) and an unnamed *Ehrlichia* sp. (1) respectively; and 32 samples were also positive with *glt*A, including *A. bovis* (10), *A. capra* (22) respectively. Of the 45 *gro*EL positive goat samples, 43 samples were positive with *rrs*, including *A. bovis* (39), *A. capra* (1) and an unnamed *Anaplasma* sp. (3), respectively; and 40 samples were positive with *glt*A, including *A. bovis* (39), *A. capra* (1) respectively.

### Phylogenetic analyses of *Anaplasma* and *Ehrlichia* species

**Figure 3:**
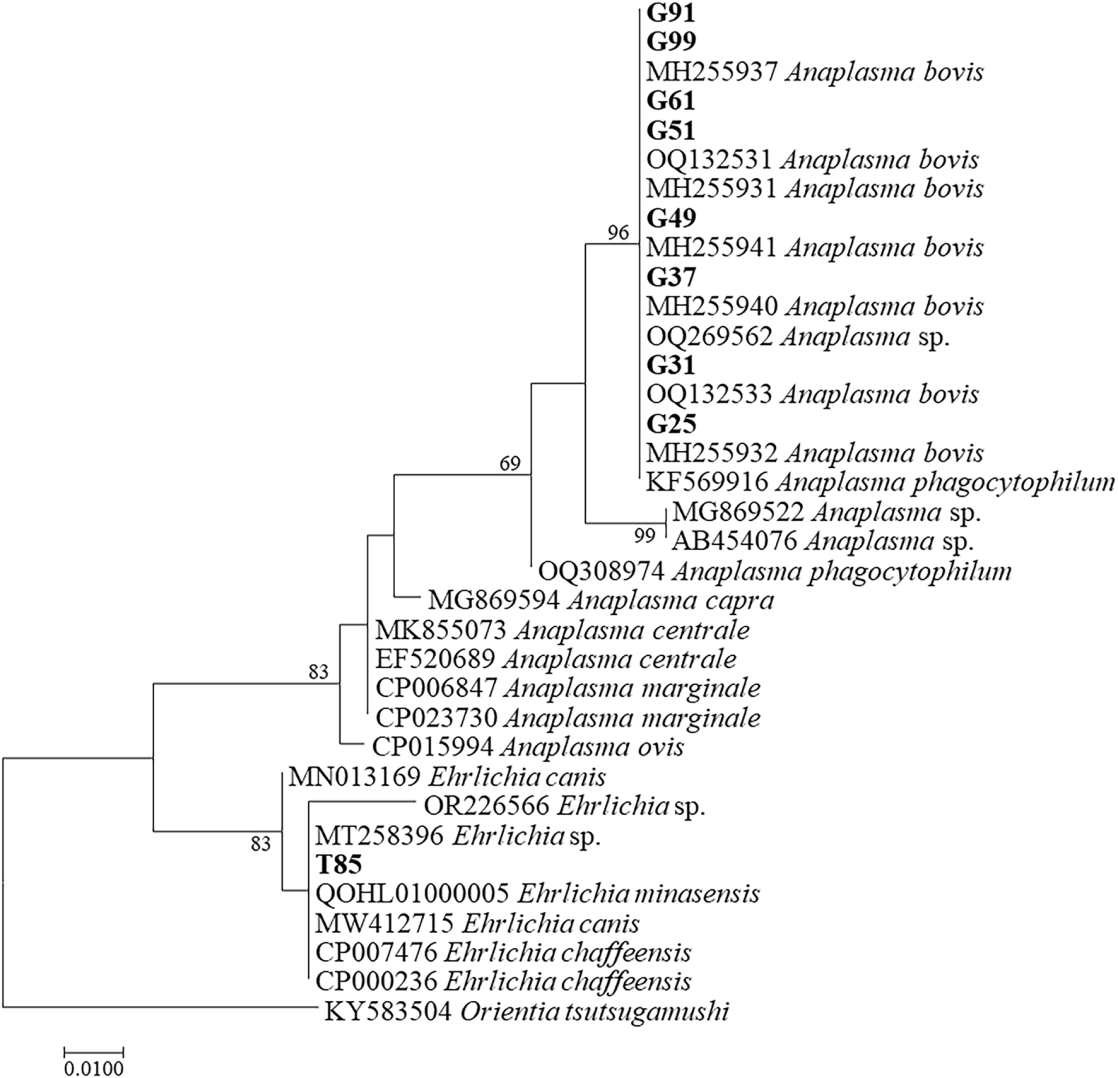
Phylogenetic relationships of the *Anaplasma* and *Ehrlichia* sequences detected from *H. longicornis* ticks and goats based on partial nucleotide sequences of the *rrs* gene (445 bp). Sequences from this study were bolded.

A total of 76 *rrs* sequences were obtained from *H. longicornis* and goats. BLAST indicated that they belonged to *A. bovis* (n = 49), *A. capra* (n = 23), an unnamed *Anaplasma* species (n = 3), and an unnamed *Ehrlichia* species (Additional file 1: Table S1). Representative strains of each species from this study were used for phylogenetic analysis. Phylogenetic analysis indicated that the sequences from goats in this study were most closely related to *A. bovis* (represented by G25, G31, G37, G49, G51, G61, G91 and G99) and that a sequence from ticks were clustered together with *E. chaffeensis* (T85) (Fig. 3).

**Figure 4:**
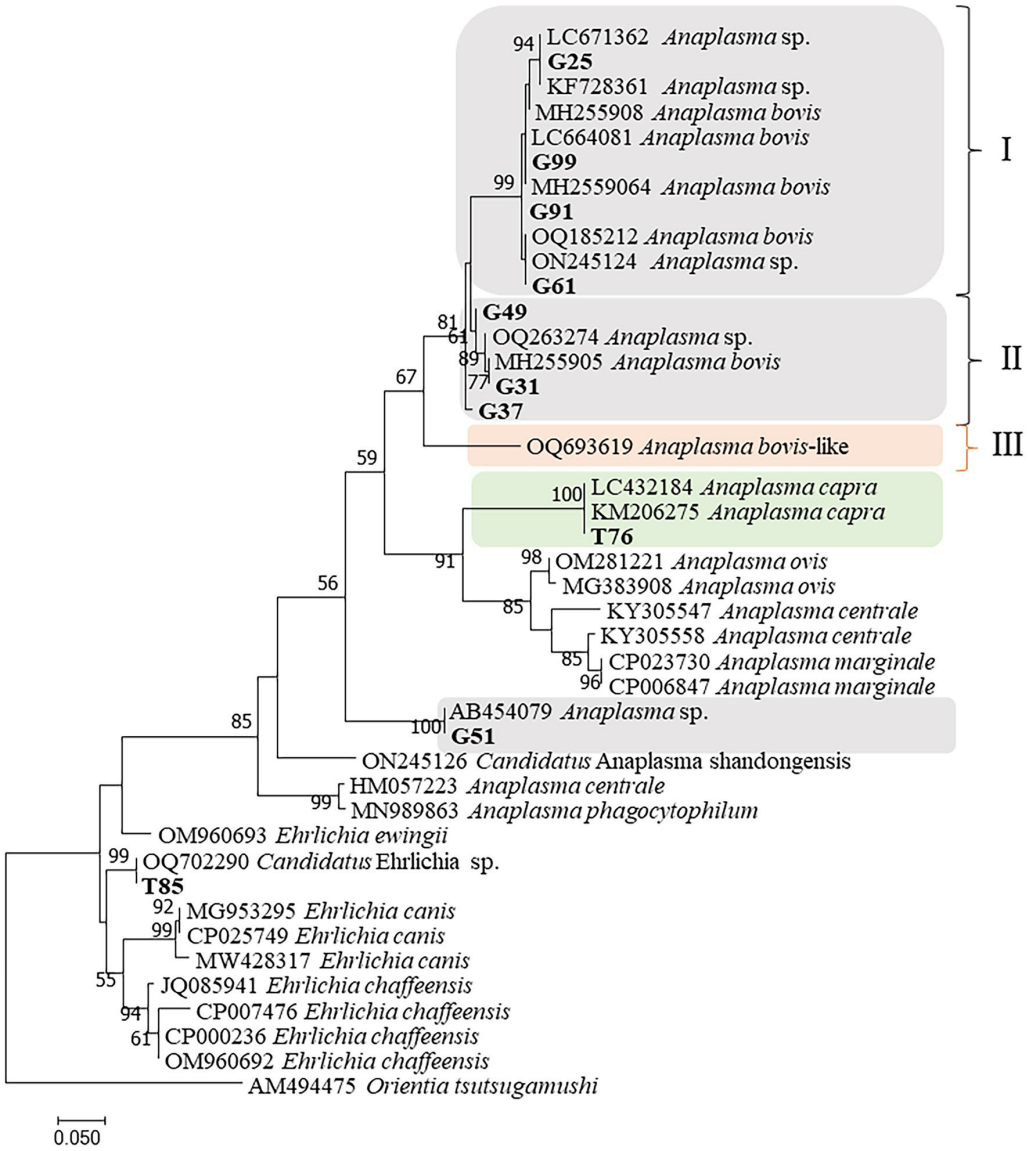
Phylogenetic relationships of the *Anaplasma* and *Ehrlichia* sequences detected from *H. longicornis* ticks and goats based on partial nucleotide sequences of the *gro*EL gene (264 bp). Sequences from this study were bolded.

The phylogeny of these strains were further analyzed with *gro*EL gene, which indicated that *Anaplasma* strains from goats belonged to 3 clusters (the gray background) (Fig. 4). Strain (G51) was in the clade with an unnamed *Anaplasma* sp. (AB454079) from deer in Japan. The strains of *A. bovis* were divided into 3 clusters ((I, II and III) and the strains of *A. bovis* from this study were dispersed in first 2 genotypes (I and II). Genotype I included strains from this study (G25, G61, G91, and G99) and previous reported *A. bovis* sequences from China (MH255906 cattle) and (ON245124 *H. longicornis*); and Genotype II included strains from this study (G31, G37, and G49) and previous reported sequences from goats in China (MH255905 and OQ263274). The third group included only the *A. bovis* strain isolated from patients in the United States (OQ693619)^22^. The *gro*EL sequence homology among the first two genotypes of *A. bovis* ranged from 92.28 to 93.75%, which is much low than the sequence homology within each group from 98.02 to 99.26%. The *gro*EL phylogeny indicated that two sequences from ticks belonged to *A. capra* (T76) and *E. chaffeensis* (T85), respectively.

**Figure 5:**
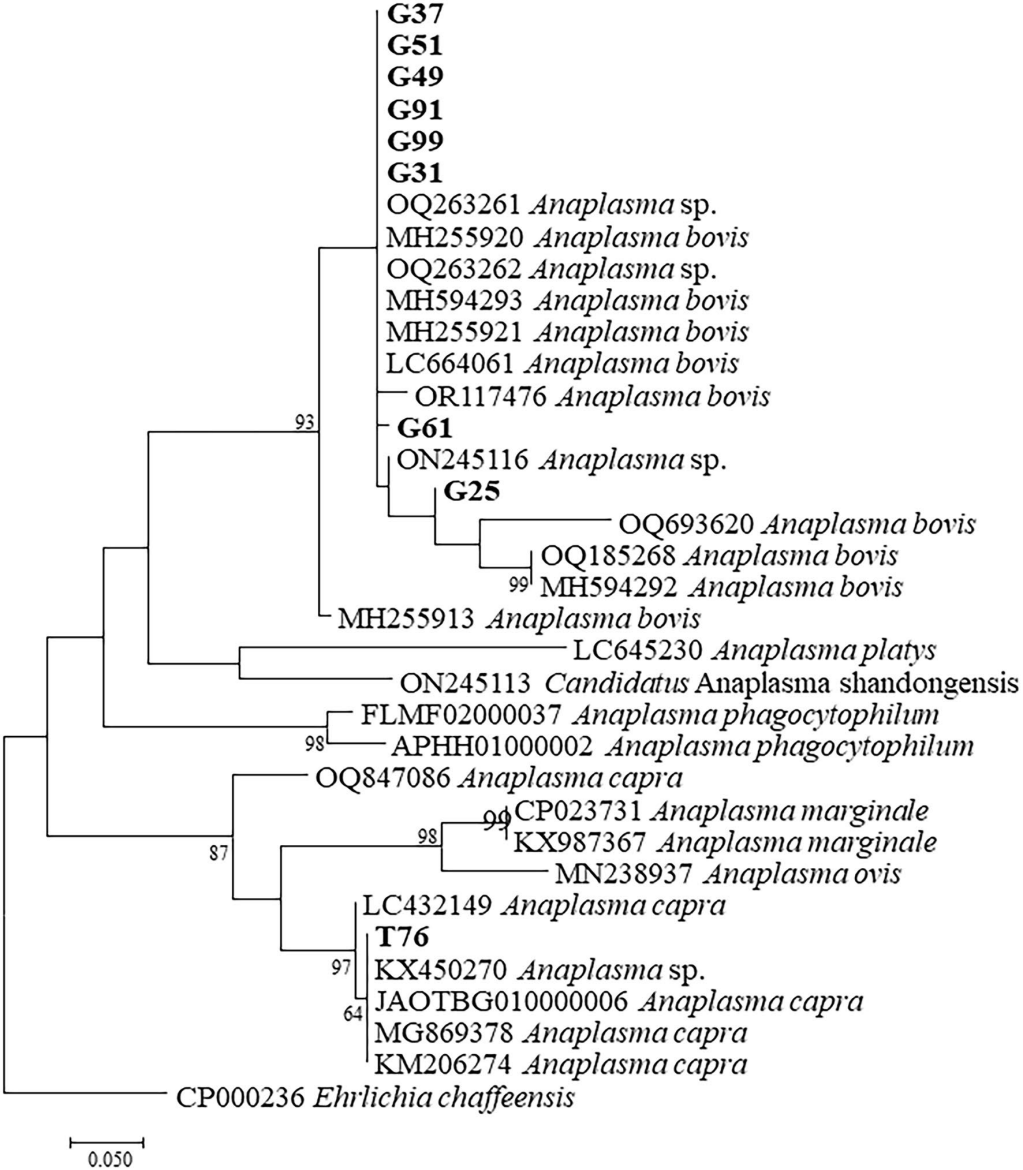
Phylogenetic relationships of the *Anaplasma* sequences detected from *H. longicornis* ticks and goats based on partial nucleotide sequences of the *glt*A gene (804 bp). Sequences from this study were bolded.

Phylogenetic analysis with *glt*A sequences showed that the strains from goats including G25, G31, G37, G49, G51, G61, G91 and G99 were all in the same clade with *A. bovis*. The tick strain T76 is in the same clade with *A. capra* (KM206274) from humans in northern China (Fig. 5).

**Figure 6:**
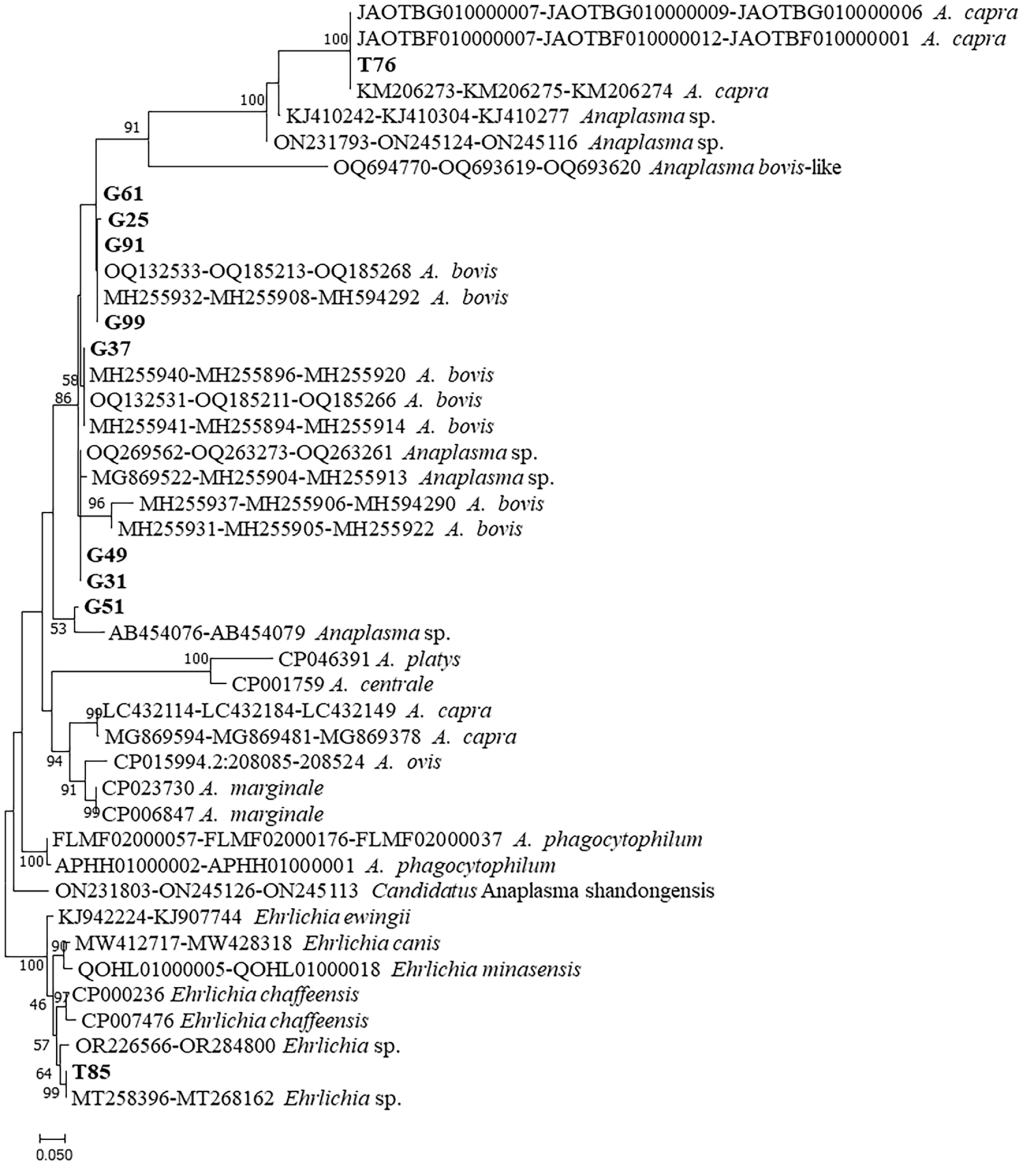
Phylogenetic tree of *Anaplasma* based on the concatenated sequences of *rrs*-*gro*EL-*glt*A (1539 bp) and *Ehrlichia* based on the concatenated sequences of *rrs*-*gro*EL (645 bp). Sequences from this study are bolded.

To get accurate phylogenetic analysis, the *rrs*, *gro*EL and *glt*A gene sequences of each strain was concatenated to do phylogeny (Fig. 6). Strain G51 was clustered together with an unnamed *Anaplasma* species (AB454079) from deer in Japan. Two tick strains from this study were classified as *A. capra* (T76) and *Ehrlichia* species (T85).

## Discussion

Suizhou County is characterized by dense scrub forests and a wide variety of wildlife species, which are suitable for ticks to reproduce, making it a landform with a high incidence of zoonotic diseases. The *H. longicornis* is one of the dominant tick species in East Asian countries. It has been reported in all provinces (except for Hong Kong and Macao Special Administrative Regions) in China^26^. This study shows that *H. longicornis* is the dominant species in Suizhou County (99.53%). *Haemaphysalis longicornis* also transmits the largest number of pathogens, with up to 44 known pathogen species, which is of great significance for the study of tick-borne pathogens^26–28^. The biological carriers of *Anaplasma* are hard ticks, among which *H. longicornis* plays a crucial role as a carrier in the transmission of *Anaplasma* pathogens.

*Anaplasma bovis* has a wide host range, including *H. longicornis*, *Rhipicephalus appendiculatus*, and *H. qinghaiensis*, and the MIR of *A. bovis* in *H. longicornis* in this study was 1.11%. Related studies have been reported in South Korea (0.1%), Shandong (1.6%), Jilin and Heilongjiang (0.3%), and Henan (9.0%) provinces of China^29–32^. Domesticated ruminants infected with *A. bovis* are mainly found in African and Asian countries^33^. The results of the current study showed that goats in Suizhou County were mainly infected with *A. bovis*, which is consistent with the results of Liu et al. in central and southern China^14^. The average prevalence of *A. bovis* infection was found to be 16.00% in goats collected in four provinces of China in 2010, with the highest prevalence up to 20.30%^14^. In Guizhou Province, the prevalence of *A. bovis* infection in goats was 30.80%^34^. The prevalence of *A. bovis* infection in goats, sheep, cattle, and deer ranged from 4.10 to 24.40%^35–38^, and even 45.71% in yak^38^. In contrast, the infection rate of *A. bovis* in ticks in China mostly ranged from 0.10 to 11.10%^35,39–41^. In this study, the infection rate of *A. bovis* in goats was 26.31% and in *H. longicornis* was 1.11%. It has been noted that *A. ovis* are persistently infected in sheep for up to 4–6 years; *Anaplasma capra* are also persistently or chronically infected in deer^42^. Because of the high infection rate of *A. bovis* in goats’ whole blood samples in this study, we hypothesized that persistent infection with *A. bovis* may exist in goats in this region.

*Anaplama bovis* was only considered as human pathogen until recently. A total of 7 cases of human infection with *A. bovis* have been reported worldwide, with 2 cases reported in Jiangxi, China in 2013 and 1 case reported in Anhui, China in 2021. In addition, 4 cases of *A. bovis*-like infection were detected in the United States from 2015 to 2017^22,43,44^. Most animals infected with *A. bovis* are asymptomatic, but human infections with *A. bovis* can present with fever (up to 39 °C), rigor, headache, myalgia, anorexia, rash, chill, diarrhea, thrombocytopenia and lymphadenopathy. Therefore, the *A. bovis* should be considered to be pathogenic to humans.

*Anaplasma capra* was first identified in goats and subsequently recognized as an emerging human pathogen in China^9^. In this study, for the first time, *Anaplasma capra* were found in *H. longicornis* collected from Hubei Province. In the present study, both *H. longicornis* (1.32%) and goats (1.31%) were infected with *A. capra*. The primary host of *A. capra* is small ruminants, and *A. capra* has been detected in 14%-18% of small ruminants^45^. *Anaplasma capra* has been found in *H. longicornis* in Shandong Province, in *H. qinghaiensis* in Gansu Province, and in a variety of tick species in South Korea^46^. *Anaplasma capra* has also been detected in *R. microplus* in the Hubei Province^47^. Sequence homology between the *A. capra* sequences in this study and those of *A. capra* isolated from patients from northern China was 100% on both *gro*EL and *glt*A. Patients infected with *A. capra* usually present with nonspecific febrile symptoms such as fever, malaise, headache, dizziness, myalgia, and chills, and some patients present with severe symptoms such as encephalitis. It is reported that a more frequent symptom in patients infected with *A. capra* than in human granulocytic anaplasmosis cases is a rash^9^.

The G51 strain in this study and the *Anaplasma* strain from Japan (AB454079) are very different from other *Anaplasma* strains and should be a new species. Ticks and domesticated animals infected with *Ehrlichia* were low in this study. The unnamed *Ehrlichia* species identified in this study was reported to be prevalent in *H. longicornis* in Hebei Province (1.2%)^48^. *Anaplasma* and *Ehrlichia* were not detected in *R. microplus*, cattle and dogs, probably due to small sample sizes. Consequently, the *Anaplasma* and *Ehrlichia* species in ticks and animals need to be further investigated in other areas in Hubei Province.

In Suizhou County, patients with unknown fever and a history of tick bites are often diagnosed and local hospitals and the local Center for Disease Control and Prevention (CDC) had difficulty to make accurate diagnoses due to lack of pathogen information. This sentinel and pioneer study in analysis of the tick borne-pathogens in the region provide knowledge of the tick-borne pathogens for physicians and health-care workers for correct diagnosis of the patients with potential tick-borne diseases in the area.

## Conclusions

Our study shows that ticks in Hubei Province carry multiple *Anaplasma* species and an *Ehrlichia* species, and the infection rate of *A. bovis* is relatively high in goats. *Anaplama bovis* was only considered as animal pathogen before, but recent studies showed that it is an emerging human pathogen. Therefore, human infection of *Anaplasma* and *Ehrlichia* in central China should be monitored.

### Supplementary Information

Supplementary Table S1.

## Data Availability

The sequences of this study are available in the GenBank under the accession numbers: OR647343-OR647344, OR751938-OR751939 (16S rRNA), OR671916-OR671922, OR757467-OR757469 (*gro*EL), OR671915, OR727627-OR727634 (*glt*A). (https://www.ncbi.nlm.nih.gov/nuccore).
