# Supplementary material for: Prevalence and genetic diversity of Anaplasma and Ehrlichia in ticks and domesticated animals in Suizhou County, Hubei Province, China
Source: Sci Rep. 2024 Jun 1;14:12621. doi: 10.1038/s41598-024-63267-6 (PMC11144266; doi:10.1038/s41598-024-63267-6)
Supplement: Supplementary file 1 — Supplementary Table S1. [file 41598_2024_63267_MOESM1_ESM.pdf]

**Title:** Prevalence and genetic diversity of *Anaplasma* and *Ehrlichia* in ticks and domesticated animals in Suizhou County, Hubei Province, China

**Authors:**

Ju Tang[a#], Jiao Xu[a#], Xiao-hui Liu[b#], Fang-zhi Lv[b], Qiu-ju Yao[b], Xiao-Fan Zhou[a], Hui-ya Lu[a], Tian-mei Yu[a], Ze-Zheng Jiang[a], Xiao-zhou Jin[b*], Fang Guo[b*], Xue-jie Yu[a*]

**Additional file 1: Table S1.** *Anaplasma*-positive ticks and goats in Suizhou County, Hubei Province, China.

| Sampling date | Sample species | Sample ID | *rrs* | *gro*EL | *glt*A |
|---|---|---|---|---|---|
| May 2023 | *Haemaphysalis longicornis* | 21 | *Anaplasma capra* | *Anaplasma capra* | *Anaplasma capra* |
| | | 22 | *Anaplasma bovis* | *Anaplasma bovis* | *Anaplasma bovis* |
| | | 25 | *Anaplasma bovis* | *Anaplasma bovis* | *Anaplasma bovis* |
| | | 27 | *Anaplasma bovis* | *Anaplasma bovis* | *Anaplasma bovis* |
| | | 28 | *Anaplasma bovis* | *Anaplasma bovis* | *Anaplasma bovis* |
| | | 34 | - | *Anaplasma bovis* | - |
| | | 42 | - | *Anaplasma bovis* | - |
| | | 49 | *Anaplasma capra* | *Anaplasma capra* | *Anaplasma capra* |
| | | 50 | *Anaplasma capra* | *Anaplasma capra* | *Anaplasma capra* |
| | | 51 | - | *Anaplasma bovis* | - |
| | | 59 | *Anaplasma capra* | *Anaplasma capra* | *Anaplasma capra* |
| | | 66 | *Anaplasma bovis* | *Anaplasma bovis* | *Anaplasma bovis* |
| | | 69 | *Anaplasma capra* | *Anaplasma capra* | *Anaplasma capra* |
| | | 71 | - | *Anaplasma bovis* | *Anaplasma bovis* |
| | | 78 | - | *Anaplasma bovis* | - |
| | | 85 | *Anaplasma capra* | *Anaplasma capra* | *Anaplasma capra* |
| July 2023 | *Haemaphysalis longicornis* | 10 | *Anaplasma capra* | *Anaplasma capra* | *Anaplasma capra* |
| | | 13 | *Anaplasma capra* | *Anaplasma capra* | *Anaplasma capra* |
| | | 18 | *Anaplasma bovis* | *Anaplasma bovis* | - |
| | | 19 | *Anaplasma capra* | *Anaplasma capra* | *Anaplasma capra* |
| | | 23 | *Anaplasma bovis* | *Anaplasma bovis* | - |
| | | 31 | - | *Anaplasma bovis* | *Anaplasma bovis* |
| | | 36 | - | *Anaplasma bovis* | *Anaplasma bovis* |
| | | 54 | - | *Anaplasma bovis* | - |
| | | 58 | *Anaplasma bovis* | *Anaplasma bovis* | - |
| | | 69 | - | *Anaplasma capra* | *Anaplasma capra* |
| | | 70 | - | *Anaplasma capra* | *Anaplasma capra* |
| | | 71 | *Anaplasma capra* | *Anaplasma capra* | *Anaplasma capra* |
| | | 72 | *Anaplasma capra* | *Anaplasma capra* | *Anaplasma capra* |
| | | 73 | *Anaplasma capra* | *Anaplasma capra* | *Anaplasma capra* |
| | | 74 | *Anaplasma capra* | *Anaplasma capra* | - |
| | | 75 | *Anaplasma capra* | *Anaplasma capra* | - |
| | | 76 | *Anaplasma capra* | *Anaplasma capra* | *Anaplasma capra* |

| Sampling date | Sample species | Sample ID | *rrs* | *gro*EL | *glt*A |
|---|---|---|---|---|---|
| | | 77 | *Anaplasma capra* | *Anaplasma capra* | *Anaplasma capra* |
| | | 78 | *Anaplasma capra* | *Anaplasma capra* | *Anaplasma capra* |
| | | 79 | *Anaplasma capra* | *Anaplasma capra* | *Anaplasma capra* |
| | | 80 | *Anaplasma capra* | *Anaplasma capra* | *Anaplasma capra* |
| | | 81 | *Anaplasma capra* | *Anaplasma capra* | *Anaplasma capra* |
| | | 82 | *Anaplasma capra* | *Anaplasma capra* | *Anaplasma capra* |
| | | 83 | *Anaplasma capra* | *Anaplasma capra* | *Anaplasma capra* |
| | | 84 | - | *Anaplasma capra* | - |
| | | 89 | - | *Anaplasma bovis* | - |
| | | 90 | - | *Anaplasma bovis* | - |
| | | 97 | *Anaplasma bovis* | *Anaplasma bovis* | *Anaplasma bovis* |
| | | 99 | *Anaplasma bovis* | *Anaplasma bovis* | *Anaplasma bovis* |
| | | 120 | - | *Anaplasma bovis* | - |
| August 2023 | Goat | 1 | *Anaplasma bovis* | *Anaplasma* sp. | *Anaplasma bovis* |
| | | 3 | *Anaplasma bovis* | *Candidatus* Anaplasma | *Anaplasma bovis* |
| | | 6 | | | |
| | | 14 | *Anaplasma bovis* | *Anaplasma bovis* | *Anaplasma bovis* |
| | | 17 | - | *Anaplasma capra* | - |
| | | 20 | *Anaplasma bovis* | *Anaplasma bovis* | *Anaplasma bovis* |
| | | 23 | *Anaplasma bovis* | *Anaplasma bovis* | *Anaplasma bovis* |
| | | 25 | *Anaplasma* sp. | *Anaplasma* sp. | - |
| | | 31 | *Anaplasma* sp. | *Anaplasma bovis* | - |
| | | 33 | *Anaplasma bovis* | *Anaplasma bovis* | *Anaplasma bovis* |
| | | 34 | *Anaplasma bovis* | *Anaplasma bovis* | *Anaplasma bovis* |
| | | 36 | *Anaplasma bovis* | *Anaplasma bovis* | *Anaplasma bovis* |
| | | 37 | *Anaplasma bovis* | *Anaplasma bovis* | *Anaplasma bovis* |
| | | 44 | *Anaplasma bovis* | *Anaplasma bovis* | *Anaplasma bovis* |
| | | 45 | *Anaplasma bovis* | *Anaplasma bovis* | *Anaplasma bovis* |
| | | 46 | *Anaplasma bovis* | *Anaplasma bovis* | *Anaplasma bovis* |
| | | 47 | *Anaplasma bovis* | *Anaplasma bovis* | *Anaplasma bovis* |
| | | 48 | *Anaplasma bovis* | *Anaplasma bovis* | *Anaplasma bovis* |
| | | 49 | *Anaplasma bovis* | *Anaplasma bovis* | *Anaplasma bovis* |
| | | 51 | *Anaplasma bovis* | *Anaplasma* sp. | - |
| | | 52 | *Anaplasma bovis* | *Anaplasma bovis* | *Anaplasma bovis* |
| | | 53 | *Anaplasma bovis* | *Anaplasma bovis* | *Anaplasma bovis* |
| | | 54 | *Anaplasma bovis* | *Anaplasma bovis* | *Anaplasma bovis* |
| | | 55 | *Anaplasma bovis* | *Anaplasma bovis* | *Anaplasma bovis* |

| Sampling date | Sample species | Sample ID | *rrs* | *gro*EL | *glt*A |
|---|---|---|---|---|---|
| | | 58 | *Anaplasma bovis* | *Anaplasma bovis* | *Anaplasma bovis* |
| | | 59 | *Anaplasma bovis* | *Anaplasma bovis* | *Anaplasma bovis* |
| | | 61 | *Anaplasma bovis* | *Anaplasma bovis* | *Anaplasma bovis* |
| | | 62 | *Anaplasma bovis* | *Anaplasma bovis* | *Anaplasma bovis* |
| | | 63 | *Anaplasma bovis* | *Anaplasma bovis* | *Anaplasma bovis* |
| | | 69 | *Anaplasma bovis* | *Anaplasma bovis* | *Anaplasma bovis* |
| | | 71 | *Anaplasma bovis* | *Anaplasma bovis* | *Anaplasma bovis* |
| | | 75 | *Anaplasma bovis* | *Anaplasma bovis* | *Anaplasma bovis* |
| | | 78 | *Anaplasma bovis* | *Anaplasma bovis* | *Anaplasma bovis* |
| | | 80 | *Anaplasma bovis* | *Anaplasma bovis* | *Anaplasma bovis* |
| | | 81 | *Anaplasma bovis* | *Anaplasma bovis* | *Anaplasma bovis* |
| | | 87 | *Anaplasma bovis* | *Anaplasma bovis* | *Anaplasma bovis* |
| | | 90 | *Anaplasma bovis* | *Anaplasma bovis* | *Anaplasma bovis* |
| | | 91 | *Anaplasma bovis* | *Anaplasma bovis* | *Anaplasma bovis* |
| | | 98 | *Anaplasma bovis* | *Anaplasma bovis* | *Anaplasma bovis* |
| | | 99 | *Anaplasma* sp. | *Anaplasma bovis* | *Anaplasma bovis* |
| | | 106 | *Anaplasma capra* | *Anaplasma capra* | *Anaplasma capra* |
| | | 107 | *Anaplasma bovis* | *Anaplasma bovis* | *Anaplasma bovis* |
| | | 108 | *Anaplasma bovis* | *Anaplasma bovis* | *Anaplasma bovis* |
| | | 112 | *Anaplasma bovis* | *Anaplasma bovis* | *Anaplasma bovis* |
| | | 115 | - | *Anaplasma bovis* | - |
